# Death of Dr. Kane

**Published:** 1857-04

**Authors:** 


### EDITORIAL.

*Death of Dr. Kane.*

We are sure our readers will all be interested in the following account of the sickness and death of Dr. E. K. Kane, which we copy from the *Boston Medical and Surgical Journal*:—

'The death of the late Dr. E. K. Kane, which took place in Havana on the 16th of February, though not unexpected, has still filled the minds of all who knew him with deep regret that a career so brilliantly commenced and so faithfully followed, should be so prematurely terminated. It was the fortune of the writer to attend him, in consultation with his regular physician (Dr. La Riverend), during the last part of the sickness which terminated his life. A few particulars of his case, gathered in that short period, will, it is believed, derive some interest from their connection with one so justly celebrated.

'Dr. Kane inherited a decided predisposition to rheumatic affection, and had from early life been subject to attacks of articular rheumatism. He suffered very severely from this disease after his return from the first Arctic expedition. The heart, also, had become involved, and he was thought to have a considerable degree of hypertrophy, together with thickening of the valves. So severely was he afflicted with articular rheumatism while preparing for the last cruise in search of Sir John Franklin, that it was often necessary to apply frictions to the joints for an hour, before rising in the morning, in order to enable him to ride to the Navy Yard, where the "Advance" was fitting out.

'Very soon after getting into the high latitudes, however, these difficulties subsided—a result which would hardly have been anticipated, but which he had observed in his own case on his previous voyage. What his sufferings and exposures were during his Artic expedition, is well known; but it is proper to state that they were much more severe, and their effect upon his constitution more disastrous than would be supposed, from the few allusions made to his own case in the published account of the expedition.

'On his return, his previous rheumatic and cardiac troubles had become complicated with scurvy; though very much exhausted and worn out by the hardships he had undergone, he allowed himself no time for repose, but labored incessently in preparing the account of his expedition for publication. This fatigue, together with the great change in climate and habits, brought on a severe relapse of his constitutional disease, aggravated by the newly acquired scorbutic taint. He received little or no benefit from the treatment of his disease while in this country, and was advised to try a change of climate; accordingly after the publication of his book, he sailed for England. Here his health became much better, all his symptoms were much improved, and he considered himself nearly restored to health. As, however, there still remained some traces of scurvy about him, his physicians advised him to spend the winter in the West Indies, for the benefit of the climate and fruits.

'Since his return from the north, there was a somewhat remarkable change in his ability to bear the motion of the ship; he had become unusually sensitive to sea-sickness, which was brought on by even a slight roll of the vessel. The voyage from London to St. Thomas was, however, well supported; while there his health continued to improve, and at the end of six weeks he sailed for Havana. The ship in which he took passage was overtaken by a severe storm; he was very much affected by the motion of the vessel, and in the effort and strain of vomiting ruptured a blood-vessel in the brain. Entire insensibility followed, and continued for several days after his arrival in Havana. A partial recovery took place after a few days, but the right side was found to be completely paralyzed.

'During the months of December and January and until the 10th of February, he slowly rallied from this attack, and was able to walk a little about his room and to drive out. He recovered the use of the right hand and wrist to a great degree, and shortly before the second attack was able to rotate the fore-arm. His mind was perfectly clear, although there was some loss of control over the memory. When he endeavored to recal any circumstance which had transpired, several others, more or less connected with it, were remembered, from which he was unable to isolate the particular fact desired. Of this difficulty he was himself perfectly conscious.

'On the 10th of February, at the morning visit, he appeared more cheerful than usual, and conversed a good deal with those about him. About 11 o'clock, however, he was suddenly seized with a severe attack of apoplexy, which deprived him entirely of consciousness. There was at first considerable spasmodic action of the muscles, which simulated in some degree a fit of epilepsy. These symptoms soon subsided, leaving him with almost complete paralysis of the entire body. The iris responded to light, and the muscles of the pharynx acted when stimulated by fluid introduced into the mouth. The pulse was feeble, and varied from one hundred and twenty to one hundred and forty beats. The skin was moist and cool. He remained very much in this state until his death, which took place on the fifth day after the seizure. In this interval, however, he seemed to have recovered some degree of consciousness, and several times signified assent to a question by turning his eyes toward the speaker. There was some motion of the lips when a spoon was placed in the mouth, and once or twice he was able to make sensible pressure with the right hand. There was no indication of suffering during his last hours, and he died apparently from simple exhaustion.

'The tenacity of life in this case was quite remarkable. A constitution broken by chronic disease of many years' standing —a series of hardships and exposures almost unheard of, with all the depressing addition of care and responsibility—followed by an affection which for some months threatened his life; add to all these an attack of apoplexy, paralyzing entirely the right side, and in two months after a relapse affecting the whole body, and one can hardly conceive how life could have been sustained for so long a period as five days after the last shock.

'The treatment in this case was quite simple. On acconnt of his previous illness and the scorbutic taint in his system, it was thought unsafe to resort to the active measures usually pursued in such cases. After the first attack, small doses of ext. nux vomica with quinine were administered. These were suspended after a time, through fear of increasing the cardiac disease, and a high tonic and anti-scorbutic course was followed. After the second attack, a few leeches were applied, together with cold applications to the head. F. S. AINSWORTH.'

